# Major Spinal Surgery Between Two Documented COVID-19 Infections in an Elderly Female: A Case Report

**DOI:** 10.31729/jnma.6507

**Published:** 2021-10-31

**Authors:** Bishruti Sharma, Nabin Paudyal, Binod Rajbhandari, Amit Pradhanang, Nikita Dwa, Ajay Pradhan, Mohan Raj Sharma

**Affiliations:** 1 Department of Neurosurgery, Chirayau National Hospital and Medical Institute, Basundhara, Kathmandu, Nepal; 2 Department of Internal Medicine, Chirayau National Hospital and Medical Institute, Basundhara, Kathmandu, Nepal; 3 Department of Surgery, Nobel Medical College and Teaching Hospital, Biratnagar, Nepal

**Keywords:** *COVID-19*, *re-infection*, *spinal fracture*

## Abstract

Documented re-infection of COVID-19 is uncommon and doing a major spinal surgery in an elderly patient right after the recovery from the first event is itself a major undertaking. Re-infection after successful surgery points to the possibility of COVID-19 infection being a post-surgical complication. Here, we report a case of a 72-years-old elderly female who had presented to us with features of COVID-19 infection confirmed by reverse transcription polymerase chain reaction assay and unstable spinal fracture who underwent a pedicle screw fixation for the fracture of the third and fourth thoracic vertebrae after two consecutive negative serology assays. A month after discharge from the hospital, she presented with severe symptoms of COVID-19 again confirmed by two consecutive polymerase chain reaction assays. She was managed conservatively and was discharged without significant respiratory and neurological complications. We described this case in detail in addition to reviewing the pertinent literature.

## INTRODUCTION

Documented re-infection of COVID-19 is uncommon and is reported between 10-15%.^1^ With increasing occurrence of asymptomatic infections of the disease, as much as 40% may have re-infection.^1^ To do a major spinal surgery in a frail and elderly patient right after recovery from COVID is itself a major undertaking. Being discharged home after successful surgery and coming back to the hospital with signs and symptoms suggestive of COVID-19 after a month of the previous infection is extremely rare.^2^ We report this unique case who was managed successfully in our hospital in addition to reviewing pertinent literature.

## CASE REPORT

**Figure 1:**
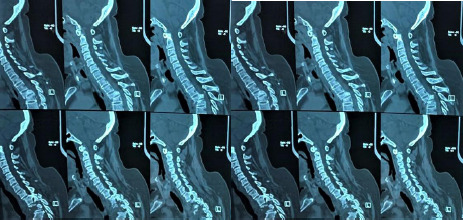
Sagittal section showing fracture of the third and fourth vertebrae compressing the spinal cord.

A 72-year-old lady with a history of fall from a height of 3meters presented to the emergency department of our hospital in October 2020, with inability to move her left leg following the trauma. The patient also had shortness of breath for 4 hours and generalized chest pain at the time of presentation. She did not have fever, cough, sweating and palpitation. She was a hypertensive and was under regular medication (Telmisartan 40 mg once daily) for 8 years. She had no other significant past medical or surgical history. She had normal psychosocial history and had no prior history of identifiable genetic disorders within her family members. Her vitals were stable. On physical examination of the respiratory system, there was bilateral equal air entry with vesicular breath sounds and no added sounds. Neurological examination revealed paraparesis of 4/5 in her right leg and 3/5 in her left leg. The rest of the physical examination was normal. The family later disclosed that the patient had tested COVID-19 positive two days before the injury and was living in isolation in her home. Polymerase chain reaction (PCR) reports documenting the COVID-19 infection showed positive serological status (open reading frame (ORF)-1 ab gene Cycle Threshold (CT) value = 34.48 and Nucleocapsid gene (N-gene) CT value = 33.08). Patient underwent routine laboratory tests and imaging studies. A non-contrast computed tomography (CT) scan of the dorsal spine revealed a comminuted unstable fracture of the T3 and T4 vertebrae (Figure 1). There was also comminuted fracture of the midshaft region of the right clavicle displaced anteriorly along with sternoclavicular joint subluxation.

The patient was kept on bed rest in a thoracolumbosacral orthosis (TLSO) brace and received treatment for COVID-19 infection as per the government of Nepal protocol.

**Figure 2:**
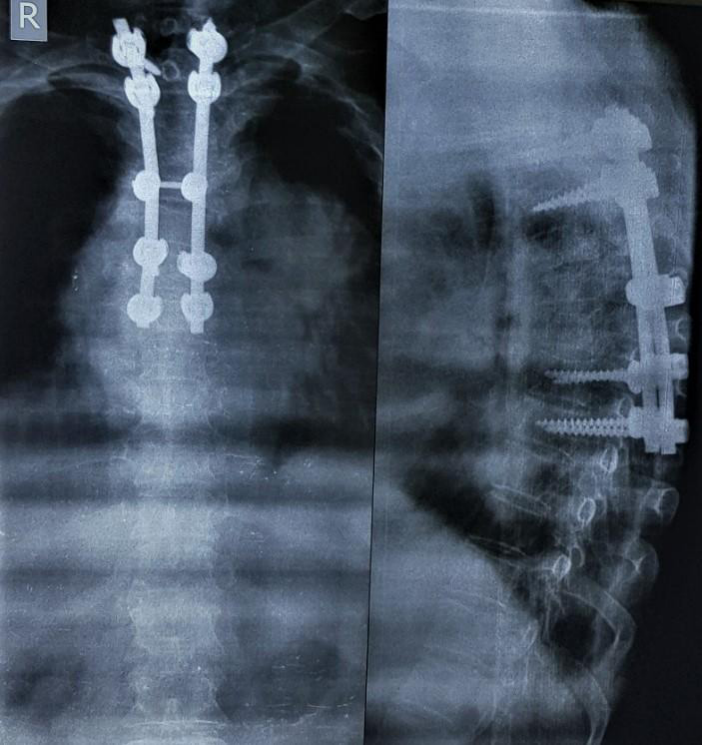
Post-operative Anteroposterior/Lateral x-ray view of dorsal spine showing long segment pedicle screw fixation of the dorsal vertebrae.

Two weeks after the admission, the patient improved to normalcy and repeated PCR for COVID-19 was negative. She was operated successfully (long segment posterior instrumentation with pedicle screws) in November 2020 (Figure 2).

The patient tolerated the surgery well. Three days after the surgery, she was mobilized in a wheelchair. Regular physiotherapy was provided and adequate dietary care was given to the patient. All events following the mobilization were satisfactory and the patient was discharged home 2 weeks after surgery.

**Figure 3:**
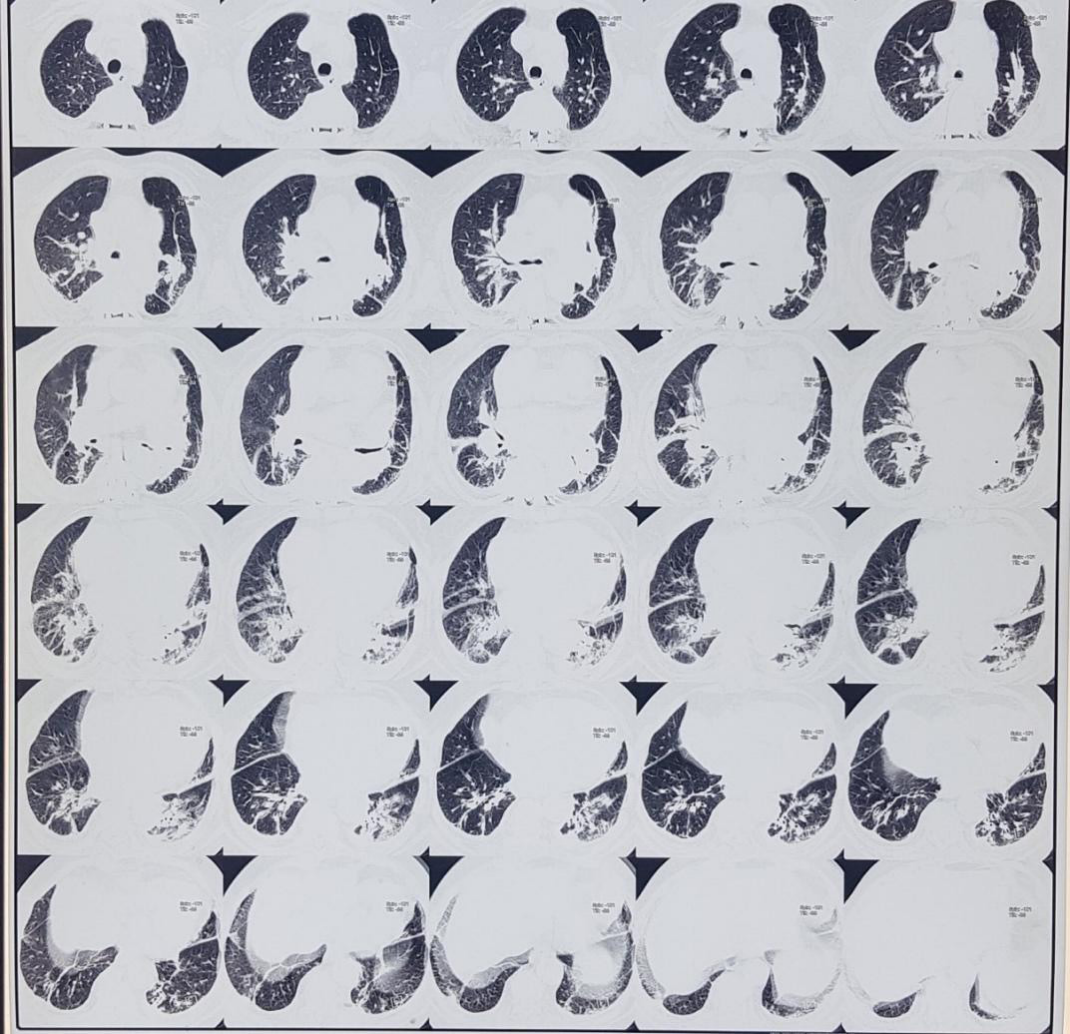
The multidetector-computed tomography scan of chest showing patchy consolidation with fibrotic bands.

One week after the discharge, she presented to our emergency department again with chief complaints of shortness of breath, productive cough and generalized chest pain for 2 days. There was no history of fever, dizziness, sweating and palpitation. Her vitals were normal except respiratory rate which was 24 breaths per minute with O2 saturation of 69% in room air which increased to 94% at 6 litres per min of O2 via face mask). On general physical examination, the patient was ill-looking. She had bilateral wheeze with fine crepitations on lower lung zones. Her ECG was normal. Her laboratory investigations including D-dimer, RT PCR for possible COVID-19 re-infection and multidetector CT-scan of chest with a pulmonary angiogram were obtained. SARS CoV2 real-time RT PCR was positive (ORF-1 ab gene CT value = 18, E-gene CT value = 18 and N-gene CT value = 20). The chest CT scan revealed patchy consolidations with fibrotic bands in bilateral lower, right middle lobe, lingular and the apical- posterior segment of the left upper lobe (Figure 3). Mild pleural effusion was noted in bilateral pleural cavities with extension along the fissure lines. These changes were compatible with COVID-19 infection.

**Figure 4:**
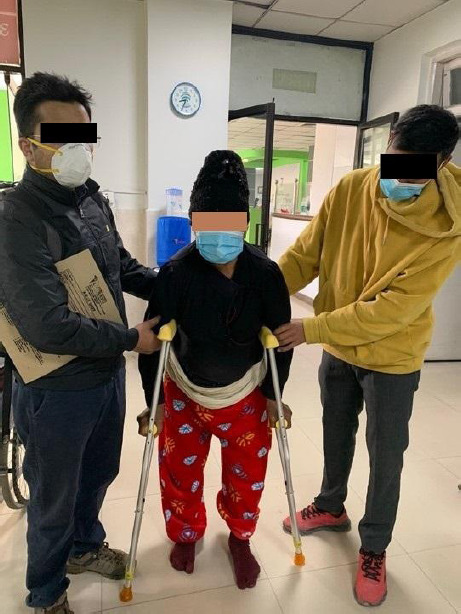
Patient at the time of follow up 2 months after surgery.

The patient was managed conservatively in the COVID ward for a few days and discharged with advice to remain in isolation until she was disease-free. A repeat PCR done 3 weeks after discharge was negative. Currently (2 months after sugery), the patient is recovering well and is ambulatory (Figure 4). She does not have significant respiratory and neurological problems and is on a regular follow up.

## DISCUSSION

Surgery leads to the alteration of the immune response. Extent of surgery, age of the patient, health status before surgery, blood transfusion and medication intake are the factors that determine the immune response. Changes in the immune response may lead to the development of post-operative infections and affect the overall recovery of the patient.^3^ COVID-19 imposes a unique risk to surgical patients. It has been reported that coronaviruses weaken the body's longterm response to the virus.^4^ As reinfections have been identified in patients with prior SARS-CoV-2 infections, the possibility of COVID-19 becoming a potential postoperative complication arises.^5^ Surgery in patients with COVID-19 infection increases perioperative complications.^6^ Hence, it is important to identify the serological status of the patients suspicious of the disease so that surgery can be safely planned. This ensures in the protection of patients and health care workers.^6^

Our study was first of its kind reporting a successful major operation between 2 consecutive COVID-19 infection. Various studies have shown that the interval between re-infection and initial infection of COVID-19 is as short as 26 days.^7-10^ In patients with clinically mild COVID-19, the immunoglobulin G (IgG) and neutralizing antibodies are detected within 2 weeks and start to decline within 30 days of the symptom onset.^7-10^ Currently, it is unknown whether every infected patient mounts a protective immune response against the virus and how long any protective impact will last.^11^ In our case, the patient presented to our hospital with features of re-infection after 28 days. Although her SARS-CoV-2 antibodies were negative, we are unsure whether she specifically developed antibodies to the virus. But we were lucky to have the opportunity to intervene and make her spine stable so that she could be rehabilitated early.

Studies have also shown increased symptom severity during the re-infection. Viral load, greater virulence and antibody-dependent enhancement are some major factors that define the severity of a re-infection.^12-15^ Our patient also had severe symptoms compared to her first documented infection.

Our case points to the possibility of COVID-19 reinfection being a post-surgical complication. Our case highlights the fact that we should be aware of the possibility of re-infection in patients with COVID-19 infection.
